# Predicting the Cochlear Dead Regions Using a Machine Learning-Based Approach with Oversampling Techniques

**DOI:** 10.3390/medicina57111192

**Published:** 2021-11-02

**Authors:** Young-Soo Chang, Hee-Sung Park, Il-Joon Moon

**Affiliations:** 1 Department of Otorhinolaryngology-Head and Neck Surgery, Sanggye Paik Hospital, College of Medicine, Inje University, Seoul 01757, Korea; yschang83@gmail.com; 2 Communication Sciences and Disorders, James Madison University, Harrisonburg, VA 22807, USA; park29hx@dukes.jmu.edu; 3 Samsung Medical Center, Department of Otorhinolaryngology-Head and Neck Surgery, School of Medicine, Sungkyunkwan University, Seoul 06351, Korea

**Keywords:** cochlear dead region, machine learning, prediction model, oversampling method, synthetic minority oversampling technique

## Abstract

*Background and Objectives*: Determining the presence or absence of cochlear dead regions (DRs) is essential in clinical practice. This study proposes a machine learning (ML)-based model that applies oversampling techniques for predicting DRs in patients. *Materials and Methods*: We used recursive partitioning and regression for classification tree (CT) and logistic regression (LR) as prediction models. To overcome the imbalanced nature of the dataset, oversampling techniques to duplicate examples in the minority class or to synthesize new examples from existing examples in the minority class were adopted, namely the synthetic minority oversampling technique (SMOTE). *Results*: The accuracy results of the 10-fold cross-validation of the LR and CT with the original data were 0.82 (±0.02) and 0.93 (±0.01), respectively. The accuracy results of the 10-fold cross-validation of the LR and CT with the oversampled data were 0.66 (±0.02) and 0.86 (±0.01), respectively. *Conclusions*: This study is the first to adopt the SMOTE method to assess the role of oversampling methods on audiological datasets and to develop an ML-based model. Considering that the SMOTE method did not improve the model’s performance, a more flexible model or more clinical features may be needed.

## 1. Introduction

The existence of cochlear dead regions was first suggested by Brian C. J. Moore in 2000 [1]. The inner hair cells, which are the transducers of the cochlea and are responsible for converting vibration patterns on the basilar membrane into action potentials in the auditory nerve, may be non-functional over a certain region of the cochlea, leading to a loss of transduction of the auditory signal from outside the cochlea to the auditory nerve. A region of the cochlea which has lost its characteristic transducing ability is defined as a cochlear dead region (DR).

Patients with cochlear dead regions have shown poor understanding of speech in noisy conditions and report less satisfaction with hearing aids than patients with no cochlear dead regions [2,3]. To achieve better clinical outcomes, a correct differential diagnosis of cochlear dead regions is imperative to help clinicians provide the best possible care to their patients. However, it is still challenging to predict DRs in patients with hearing loss based on clinical and audiologic findings [4].

In our previous study, we adopted a machine learning (ML)-based approach to develop and validate cochlear dead region prediction models as a function of frequency [5]. ML continues to evolve with advances in computing power and the field of computer science. However, we observed some limitations in our approach; the prevalence of cochlear dead regions is about five percent in the overall data if prevalence is counted by frequency. This produces an imbalanced class distribution. The predictive power or accuracy of the model might be affected by these uneven distributions in the data. A chief problem with imbalanced classification datasets is that standard machine learning algorithms do not perform well on imbalanced datasets, because many machine learning algorithms rely upon class distribution in the training dataset to gauge the likelihood of observing examples in each class when the model is used to make predictions. Therefore, the minority class is sometimes deemed as less important than the majority class, resulting in greater attention to and better performance in the majority class.

To overcome this imbalanced dataset issue, the present study adopted oversampling techniques to duplicate examples in the minority class or to synthesize new examples from the existing examples in the minority class, and then comparing the model performance of the two different datasets: the original data and the oversampled data.

## 2. Materials and Methods

### 2.1. Subjects

Patients who visited the outpatient clinic at Samsung Medical Center between September 2010 and May 2015 and who agreed to participate were enrolled in the study. Medical records, audiology results, and threshold-equalizing noise (TEN) (HL) tests were retrospectively reviewed. Patients with any signs of acute infection were excluded. All participants provided written informed consent to participate in this study. The Institutional Review Board of Samsung Medical Center approved this study (IRB No. 2010-03-004).

We used the same dataset which included the patients who were described and enrolled in our previous study [5]. Among the dataset, we used the patients’ clinical data such as sex, age, affected ear, diagnosed cause for the hearing loss, word recognition scores (WRS), types of audiogram, and pure-tone thresholds at each of the standard frequencies. Six disease groups, which we set in our previous study (sensorineural hearing loss with unknown etiology (SNHL), sudden sensorineural hearing loss (SSNHL), vestibular schwannoma (VS), Ménière’s disease (MD), noise-induced hearing loss (NIHL), and age-related hearing loss (ARHL)) were adopted [5].

### 2.2. TEN (HL) Test

The TEN (HL) test was performed following the protocol described by Moore et al. [6]; the detailed study protocol was mentioned in our previous study [5]. For the TEN (HL) test, a pure tone and a threshold-equalizing noise were played through a CD player connected to an audiometer that was calibrated to an audio player (RCD-M75U; Samsung, Suwon, Korea). The threshold-equalizing noise was shaped so that the masked threshold of a given pure tone was the same for all frequencies from 250 to 10,000 Hz in normal-hearing subjects.

A TEN level of 10 dB above the hearing threshold at a given frequency region was selected to obtain a reliable masking effect. The TEN presentation level never exceeded more than 95 dB HL [6]. Subjects were asked to detect the introduced pure tone in the TEN. The masked threshold was increased in 2-dB increments using the modified Hughson–Westlake procedure [7]. The pure-tone and TEN thresholds were obtained at 0.5, 0.75, 1, 1.5, 2, 3, and 4 kHz. At each specific frequency, when the threshold of the test tone in the TEN was 10 dB or more above the TEN level, a cochlear dead region was diagnosed at that frequency [6]. In patients where the TEN (HL) level could not be sufficiently increased to elevate the absolute threshold by 10 dB or more, the results were considered inconclusive [8]. These patients were also included in the analysis and assessed for the purposes of this study as not having cochlear dead regions at that given frequency [7].

### 2.3. Model Development

To develop the best performing ML model, the study adopted several steps during the model’s development. The first two steps are identical to the first two in our previous study [5]. We then applied oversampling techniques to the original dataset and applied the newly synthesized oversampled data to develop the ML models that we used in the present study.

First, using recursive partitioning and regression to build a classification tree (CT), we obtained the best break point for the continuous variable. We set the minimal split number at 20. The model suggests the best break point as one that splits the population into sub-populations, and we applied this break point to the continuous variable to bag the data for the second step.

Second, the study used logistic regression (LR) to construct binary classification models. Because the aim of the model development is to achieve greater accuracy in screening for the presence or absence of cochlear dead regions, we evaluated the performance of LR models with a probability of 0.1. The theoretical bases of the LR algorithms have been described in the previous study [9,10] as have the detailed processes of the model’s development [5].

Third, we employed oversampling techniques to duplicate examples in the minority class and to synthesize new examples from examples in the minority class. The study adopted the synthetic minority oversampling technique (SMOTE) [11]. SMOTE works by selecting examples that are close together in the feature space, drawing a line between the examples in said space, and then creating a new sample at a point along that line. Specifically, a random example from the minority class was first chosen.

Fourth, the study developed the new LR and CT models with oversampled data created via SMOTE to construct classification models. The method for developing the LR and CT models were the same as with the original data.

The models were constructed and tested using R (version 3.4.4, R Foundation for Statistical Computing, http://www.r-project.org/ accessed on 15 March 2018) with the rpart and caret packages [12,13]. SMOTE was performed with the DMwR (Data Mining with R) package [14].

### 2.4. Statistical Analysis and Model Evaluation Methods

Descriptive analysis was used to evaluate the prevalence of cochlear dead regions in both the original data and the oversampled data. The ‘SNHL with unknown etiology’ group was used as the reference distribution for cochlear dead regions at each frequency. Pearson’s chi-squared test was performed to analyze the distribution differences in groups. The accuracy of each model was quantified by calculating the accuracy. A 10-fold cross-validation approach to train (nine-fold) and test (one-fold) the LR and CT models was used. The results of both the CT and the LR were described in both the original data and the oversampled data. All analyses were performed using the R software package. A two-sided *p*-value < 0.05 was considered statistically significant.

## 3. Results

A total of 555 ears from 380 patients (3770 test samples) were included in the study. The descriptive statistics of the study population are listed on our previous study [5]. After applying the SMOTE method, the sample size grew to 15,494 samples. Of those 15,494 test samples, the overall frequency-specific prevalence of cochlear dead regions was 18.14%, which was originally 6.7% on our study population. The prevalence of VS etiologies, which had the lowest prevalence among the study population, increased from 7.03% to 8.64% following application of the SMOTE method. In addition, the mean WRS value was 78.9 ± 23.8%; in the original data, the value was 82.1 ± 23.9%. Descriptive statistics of the original data and oversampled data can be found in Table 1.

**Figure 1:**
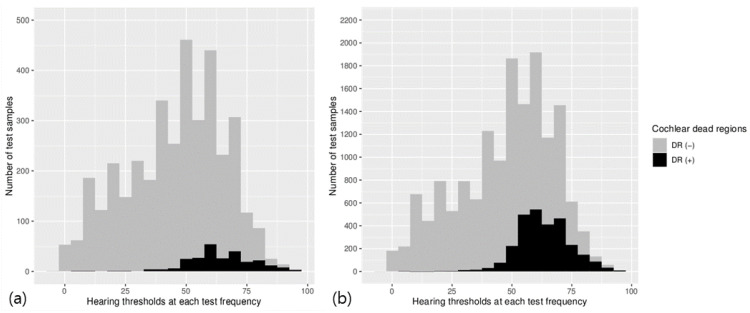
The distribution of cochlear dead regions according to the hearing thresholds at each frequency in the original data (**a**) and the oversampled data (**b**). The overall frequency-specific prevalence of cochlear dead regions was 6.7% in the original data and 18.14% in the oversampled data, respectively.

The distribution of cochlear dead regions according to the hearing thresholds at each frequency in the original data and in the oversampled data is illustrated in Figure 1. The overall proportions of the lower sample data in the original data, which indicates the frequency-specific prevalence of cochlear dead regions, were increased with the SMOTE method in the oversampled data.

**Figure 2:**
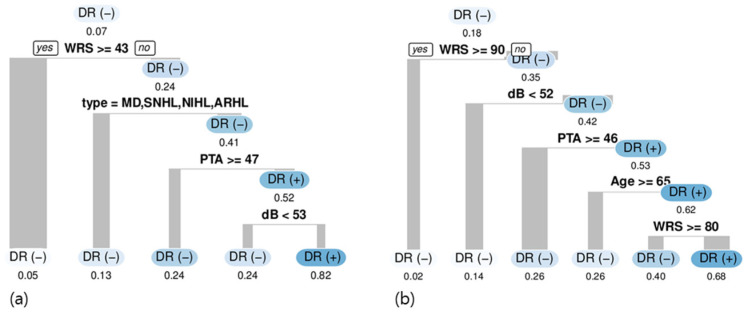
Classification tree model of the original data (**a**) and the oversampled data (**b**). DR, cochlear dead region; WRS, word recognition score; dB, decibel of at each audiometric test frequency; PTA, pure tone average of four frequencies (0.5 kHz, 1 kHz, 2 kHz, and 4 kHz); MD, Ménière’s disease; SNHL, sensorineural hearing loss; NIHL, noise-induced hearing loss; ARHL, age-related hearing loss.

The results of the CT model with the original data were described in our previous study [5]. In summary, several factors such as word recognition score (WRS) (break point: 42%), disease type (SSNHL or VS diagnosis), and average at four frequencies (0.5 kHz, 1 kHz, 2 kHz, and 4 kHz) (PTA) when higher than 47 dB (poor overall hearing threshold) were used to split the data and detect cochlear dead regions (Figure 2a). Sex, age, and side were not significantly used in the CT models.

In the CT model with the oversampled data, WRS (break point: 90%), pure-tone thresholds at each frequency (break point: 52 dB), and age were used to split the data and detect cochlear dead regions (Figure 2b). Using a WRS break point of 90%, the ratio of cochlear dead regions increased from 0.18 to 0.35, which indicates that the use of a WRS lower than 90% as a predictive factor increases predictability by a factor of two. Compared to the original data, the diagnosis of hearing loss etiologies does not increase the model’s predictive power in the oversampled data. Interestingly, for those aged under 65 with a lower WRS, a lower PTA increased the ratio of cochlear dead regions to 0.62.

The results of the multivariate logistic regression analyses for cochlear dead region detection in both the original data and the oversampled data are shown in Table 2. In the original data, VS was significantly associated with the presence of cochlear dead regions (odds ratio = 2.40, a 95% confidence interval (CI) of 1.36–4.23, *p* = 0.002), while MD showed a significantly lower odds ratio than the SNHL group (odds ratio = 0.36, 95% CI 0.18–0.73, *p* = 0.004) [5]. In the oversampled data, VS (odds ratio = 2.67, 95% CI 2.19–3.24, *p* < 0.001), SSNHL (odds ratio = 1.56, 95% CI 1.31–1.85, *p* < 0.001), and MD (odds ratio = 0.51, 95% CI 0.41–0.63, *p* < 0.001) showed a significant association with the presence of cochlear dead regions. The pure-tone thresholds of the evaluating frequencies showed a positive association with cochlear dead region presence, whereas the odds ratio for cochlear dead region presence with respect to pure-tone average was lower than the odds ratio in the control groups in both the original data and the oversampled data. Frequencies of 3000 Hz and 4000 Hz showed lower odds ratios than the reference frequency of 1000 Hz (odds ratio = 0.22, 95% CI 0.11–0.46, *p* < 0.001 and odds ratio = 0.31, 95% CI 0.15–0.62, *p* < 0.001, respectively) in the original data. In the oversampled data, all the frequencies showed more significant odds ratios than the reference frequency.

The accuracy results of the 10-fold cross-validation of the LR and CT with the original data were 0.82 (±0.02) and 0.93 (±0.01), respectively. The accuracy results of the 10-fold cross-validation of the LR and CT with the oversampled data were 0.66 (±0.02) and 0.86 (±0.01), respectively.

## 4. Discussion

The ML-based approach provided well-validated and ready-to-use prediction models for clinical practitioners. However, most ML-based classification methods tend not to perform well on minority class examples, which is common with most medical datasets. Our previous study observed the imbalanced data distribution with only 6.7% of the aimed class diagnosed as cochlear dead region. Rahman et al. proposed both an oversampling (SMOTE) and undersampling (cluster-based methods) to balance a clinical dataset [15]. They used a cardio-vascular disease dataset (823 instances and 26 attributes from the University of Hull) with two classification algorithms (the Fuzzy Unordered Rule Induction Algorithm [FURIA] and the Classification And Regression Tree [CART]) to classify the rebalanced data. The results showed the sensitivity improvement of both two classification algorithms (from 64.17% to 83.78%, FURIA and from 67.50% to 84.21%, CART). It implies that the class rebalancing technique can be applied to the clinical dataset and the performance of the class rebalancing technique depends on the ML technique used thereafter.

We investigated to overcome the imbalanced dataset issue in the present study by adopting an oversampling technique to create more evenly-distributed data. We applied oversampling techniques (SMOTE) to duplicate examples in the minority class or to synthesize new examples from examples in the minority class. SMOTE works by selecting examples that are close together in the feature space, drawing a line between those examples and extracting a new sample at a point along that line.

We produced results regarding accuracy in the 10-fold cross-validation of the LR and CT models with both the original and oversampled data: they were 0.82 (±0.02) and 0.93 (±0.01) for the original dataset and 0.66 (±0.02) and 0.86 (±0.01) for the oversampled dataset. These results indicate that the accuracy of the oversampled data was lower than that of the original data. Considering that the overall frequency-specific prevalence of cochlear dead regions was much higher than in the original data after applying the SMOTE method, this may affect the model’s accuracy with true positive data, which refers to the presence of cochlear dead regions in the present study. The hypothesis was that the machine learning models could overcome unevenly distributed data with larger clinical samples; however, the results indicate that simply applying oversampling methods did not improve the model’s performance. More powerful clinical indicators such as the audiometric configuration or type of defect when hearing a certain phoneme associated with the cochlear dead region in question may be needed to more accurately detect the presence of cochlear dead regions.

There are some limitations in our ability to detect cochlear dead regions in the general population and in increasing the applicability of these findings to clinical practice. First, TEN testing in clinics is time-consuming. Introducing more advanced technologies, such as optical coherence tomography, may reduce the time needed for testing through easily used, non-invasive methods for evaluating inner ear structures; however, the depth of the image that it produces is limited to a few millimeters due to its low permeability to tissues [16]. Therefore, the TEN (HL) test is still performed as the primary tool for assessing cochlear dead regions in clinics, although it has some limitations to being more widely used.

In addition, it is still challenging to predict the presence of cochlear dead regions in patients with hearing loss using clinical and audiologic findings [4], because the prevalence and possible indicators of cochlear dead regions differ depending on the study population [4,17,18,19]. No definitive indicators have been proven to predict the presence of cochlear dead regions in the general population. Although previous studies have revealed some reliable indicators of cochlear dead regions based on detection by TEN (HL) tests [8,20], there are only a few reports that certain hearing thresholds at each test frequency may be possible markers for cochlear dead regions. In addition, there have been no previous studies that specifically address cochlear dead region prediction as a function of frequency-specific information. For example, there are no reports on whether frequency, hearing thresholds, or etiologies of hearing loss have been weighted to address cochlear dead region prediction. Therefore, it is still unclear which patients beyond those with severe-to-profound hearing loss should undergo TEN (HL) testing, something which prevents cochlear dead regions from being more deeply integrated into clinical practice.

We address the prediction model for cochlear dead regions according to frequency and compare the results between the original data and the SMOTE oversampled data. The study results can be helpful for predicting or detecting cochlear dead regions according to frequency in clinical settings. The comparison results imply the existence of hidden associated factors or non-linearity in predicting cochlear dead regions. Previous studies have only assessed the prevalence of cochlear dead regions by ear, not by frequency. In our previous study, we assessed the prevalence of cochlear dead regions by frequency; however, the distribution of cochlear dead regions was only 6.7% and it might be suggested that an imbalanced class distribution could affect the accuracy of ML model development.

In contrast with previous studies [4,18], the feature “high frequencies” was negatively associated with cochlear dead regions in the present LR model used on the original dataset. This result may depend on the study population. Our study enrolled both ARHL and NIHL patients. These populations show a low prevalence of cochlear dead regions, despite poorer hearing thresholds at high frequencies. After adopting the SMOTE method to modify the imbalanced distribution, the results remained similar to the original data. Therefore, our results suggest that the feature “high frequencies” might be a negatively associated factor in predicting the absence of cochlear dead regions and should be considered with the diagnosed etiologies.

Because this study included patients with diverse etiologies and vastly differing levels of hearing loss, the indicators identified here may be beneficial for determining which patients have suspected cochlear dead regions. WRS, etiology type, and hearing thresholds at specific frequencies are all informative factors. WRS, which has been addressed in a previous study [21], can be a useful indicator for predicting cochlear dead regions; this was demonstrated here in this present study with the oversampled data, although the cut-off value may vary depending on the study population. In the CT model, a 43% value used for classifying the break point in WRS with the original dataset was suggested and a 90% value used for classifying the break point in WRS with oversampled data was also suggested.

The features of a disease’s etiology, such as of VS and MD, did not show any predictive power in the CT models with the oversampled data. This might be related to the use of the oversampling method, which affects the distribution according to the disease etiology and thus attenuating the predictive effects of disease etiology.

This study has some limitations. First, because the possible risk factors for cochlear dead regions are not fully understood, and the present study was performed in a retrospective manner, we could not assess all possible features. Adaptation of limited features may affect the predictive power of the ML models. Second, we applied the SMOTE technique to duplicate examples in the minority class. However, it may affect the exaggeration of unnecessary clinical features during the oversampling. This could not be assessed, because we still cannot determine which clinical features are more clinically associated and highly valuated to predict cochlear dead region. If we had selectively oversampled some features that we found in the previous study, the prediction power would have been biased with some minority classes with high predictive power, which could not be elucidated in the original dataset. Therefore, we applied the SMOTE method to observe the features of this clinical dataset.

## 5. Conclusions

This study possesses enough strength to support several conclusions. This study is the first to adopt the SMOTE method to assess the role of the oversampling method on audiological datasets and to analyze its implications for improving the prediction power of ML-based models. WRS, etiology type, and hearing thresholds at different frequencies can be suggested as potential factors for predicting cochlear dead regions. However, our results imply that simply applying the SMOTE method does not improve the model’s prediction accuracy. Further investigation for data pre-processing to apply diverse non-linear models as well as a larger sample size will be helpful to develop a more powerful and accurate model for predicting cochlear dead regions.

## Figures and Tables

**Table 1:** Comparison of clinical characteristics of the study population between the original data and the oversampled data.

	Original Data (555 Ears)	Oversampled Data (15,494 Samples)
Side		
Right	285 (51.35%)	7857 (50.71%)
Left	270 (48.65%)	7637 (49.29%)
PTA (dB)	44.8 ± 16.0	33.4 ± 13.1
WRS (%)	82.1 ± 23.9	78.9 ± 23.8
Types of diseases		
SNHL with unknown etiology	114 (20.54%)	3513 (22.67%)
SSNHL	99 (17.84%)	2649 (17.10%)
VS	39 (7.03%)	1339 (8.64%)
MD	65 (11.71%)	1832 (11.82%)
NIHL	70 (12.61%)	1882 (12.15%)
ARHL	168 (30.27%)	4279 (27.62%)

Mean pure-tone average (PTA) was calculated for four frequencies (0.5 kHz, 1 kHz, 2 kHz, and 4 kHz). WRS, word recognition score; SNHL, sensorineural hearing loss; SSNHL, sudden sensorineural hearing loss; VS, vestibular schwannoma; MD, Ménière’s disease; NIHL, noise-induced hearing loss; ARHL, age-related hearing loss.

**Table 2:** Results of multivariate logistic regression analyses for detecting cochlear dead regions.

	Original Data			Oversampled Data		
	Odds Ratio	95% Confidence Interval	*p*-Value	Odds Ratio	95% Confidence Interval	*p*-Value
Age	0.99	0.98–1.01	0.36	0.99	0.99–1.00	<0.001 *
Sex (reference: Female) Male	0.42	0.29–0.61	<0.001 *	0.52	0.48–0.61	<0.001 *
PTA (dB)	0.94	0.92–0.96	<0.001 *	0.95	0.94–0.96	<0.001 *
WRS (reference: ≥40) <40	3.77		<0.001 *	1.90	1.67–2.17	<0.001 *
Pure tone threshold of each frequency (dB)	1.11	1.09–1.13	<0.001 *	1.11	1.10–1.12	<0.001 *
Types of diseases						
(reference: SNHL)						
SSNHL	1.45	0.88–2.41	0.15	1.56	1.31–1.85	<0.001 *
VS	2.40	1.36–4.23	0.002 *	2.67	2.19–3.24	<0.001 *
MD	0.36	0.18–0.73	0.004 *	0.51	0.41–0.63	<0.001 *
NIHL	0.46	0.18–1.15	0.10	1.05	0.82–1.34	0.70
ARHL	0.96	0.53–1.74	0.88	0.88	0.73–1.07	0.21
Frequency						
(reference: 1000 Hz)						
500 Hz	1.36	0.74–2.53	0.32	0.66	0.55–0.86	<0.001 *
750 Hz	1.12	0.60–2.07	0.73	0.73	0.60–0.90	<0.001 *
1500 Hz	0.66	0.34–1.26	0.21	0.77	0.62–0.93	0.002 *
2000 Hz	0.82	0.44–1.53	0.53	0.73	0.66–0.97	0.009 *
3000 Hz	0.22	0.11–0.46	<0.001 *	0.19	0.17–0.27	<0.001 *
4000 Hz	0.31	0.15–0.62	<0.001 *	0.13	0.12–0.20	<0.001 *
Intercept	0.01	0.00–0.02	<0.001 *	0.02	0.01–0.03	<0.001 *

Mean pure-tone average (PTA) was calculated for four frequencies (0.5 kHz, 1 kHz, 2 kHz, and 4 kHz). WRS, word recognition score; SNHL, sensorineural hearing loss; SSNHL, sudden sensorineural hearing loss; VS, vestibular schwannoma; MD, Ménière’s disease; NIHL, noise-induced hearing loss; ARHL, age-related hearing loss. * *p* < 0.05.

## Data Availability

The datasets generated during and/or analysed during the current study are available from the corresponding author on reasonable request.
